# Fatty acid nitroalkenes regulate intestinal lipid absorption

**DOI:** 10.1016/j.jlr.2025.100855

**Published:** 2025-07-04

**Authors:** Francisco J. Schopfer, Lihong Teng, Ahssan Sekandari, Ese S. Ekhator, Alison B. Kohan, Bruce A. Freeman, Marco Fazzari

**Affiliations:** 1 Department of Pharmacology and Chemical Biology, University of Pittsburgh, Pittsburgh, PA; 2 Pittsburgh Heart, Lung, Blood, and Vascular Medicine Institute (VMI), Pittsburgh Liver Research Center (PLRC), Center for Immunometabolism, Pittsburgh, PA; 3 Division of Endocrinology and Metabolism, Department of Medicine, University of Pittsburgh, Pittsburgh, PA; 4 Department of Immunology, University of Pittsburgh, Pittsburgh, PA

**Keywords:** Nitro-oleic acid, nitroalkenes, electrophile, chylomicron, fat absorption, lymph flow

## Abstract

Fatty acid nitroalkenes (NO_2_-FA) are tissue-protective and anti-inflammatory endogenous lipid mediators. A unique electrophilic character promotes reversible reactions with protein thiols, influencing key cellular functions. Given their generation during digestion and therapeutic potential, understanding the mechanisms and impact of NO_2_-FA absorption and distribution is crucial. We investigated the intestinal absorption of orally administered 10-nitro-octadec-9-enoic acid (10-NO_2_-OA) in male and female rats, using a portal vein- and mesenteric lymph duct-cannulated conscious model. There were no sex-related differences in the plasma distribution of 10-NO_2_-OA and its inactive metabolite 10-nitro-octadecanoic acid (10-NO_2_-SA). 10-NO_2_-OA was extensively esterified into triglycerides at concentrations ∼60 times greater than the free acid. Duodenal administration showed that 10-NO_2_-OA is primarily incorporated into chylomicron triglycerides (TAG) and transported via the lymphatic system, bypassing initial hepatic metabolism. Notably, 10-NO_2_-OA significantly reduced lymph flow, chylomicron secretion, and impacted the lymphatic TAG profile and transit. Assessment of intestinal TAG uptake by ^3^H-triolein tracing in mice showed that 10-NO_2_-OA significantly reduced dietary fat absorption by 75%. Quantitation of radioactivity distribution along the gastrointestinal tract showed a trend to greater lipid incorporation into the mucosa. Overall, these results show that NO_2_-FA are primarily absorbed and transported through the lymphatic system as esterified TAG species, undergoing initial metabolism in enterocytes regardless of sex, with an unexpected impact on intestinal fatty acid uptake. These findings reveal novel actions of both endogenously formed and orally administered electrophilic NO_2_-FA in modulating inflammatory and metabolic syndrome-related pathologies.

## Graphical abstract

**Figure:**
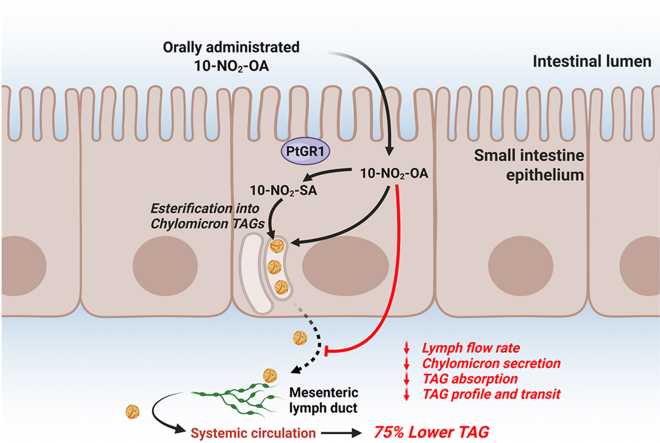

Fatty acid nitroalkenes (NO_2_-FA) are endogenous bioactive lipid mediators, with synthetic homologs being developed as therapeutic agents for treating inflammatory and metabolic-related diseases (1, 2). NO_2_-FA are detected endogenously in humans and have been reported in plants (Arabidopsis thaliana, olives) and rodents (3, 4, 5, 6, 7). Formed via nitrite (NO_2_^-^) and nitric oxide (^•^NO)-dependent reactions with primarily conjugated diene-containing fatty acids, electrophilic NO_2_-FA exhibit multi-target signaling actions and unique pharmacokinetic (PK) and pharmacodynamic (PD) profiles. In mammals, the acidic environment of the stomach serves as a key site for NO_2_^-^ conversion into a panoply of reactive species, including the potent nitrating species nitrogen dioxide (^•^NO_2_) (8). In this context, dietary conjugated dienes emerge as preferential targets for nitration by ^•^NO_2_, exceeding the reactivity of bis-allylic unsaturated fatty acids by over 10^5^-fold (3). The nitro-nitrate ester derivatives of conjugated linoleic acid have also been detected in the gastric compartment during digestion. These products can be absorbed directly or after the nitrate ester substituent dissociates, yielding an electrophilic nitroalkene in the gastrointestinal tract (9).

Beyond the gastric environment, NO_2_-FA formation has also been reported in tissues upon cardiac ischemia-reperfusion or inflammatory responses and has also been localized in mitochondria and activated macrophages. The electrophilic nature of the nitroalkene substituent promotes both rapid and reversible Michael addition reactions with a population of hyperreactive nucleophilic cysteine residues (nitroalkylation) in proteins (10), leading to conformational changes and functional alterations (11). Both preclinical and clinical data affirm that NO_2_-FA induce broad antioxidant and cytoprotective signaling responses and the resolution of inflammation and fibrosis (12, 13). Various animal models of disease, spanning from obesity-related cardiopulmonary disorders to neurodegeneration, exhibit beneficial outcomes upon administration of nitroalkenes (14, 15). Such encouraging results have motivated the clinical evaluation of NO_2_-FA as a new drug agent. Thus, the safety and the signaling pathway engagement of 10-nitro-octadec-9-enoic acid (10-NO_2_-OA), a synthetic biosimilar of the natural nitro conjugated linoleic acid, has been evaluated in 5 Phase I clinical studies encompassing intravenous and oral formulations (16, 17) and an ongoing Phase 2 trial in obesity-related asthma.

The intestinal absorption of dietary fats is a complex process that can be summarized by the following: (a) enzymatic digestion of dietary TAG into free fatty acids (FFA), glycerol and monoacylglycerols, (b) absorption by the intestinal mucosa, (c) intracellular processing and re-esterification, and TAG incorporation into apoB-48 containing TAG-rich chylomicrons and transport by the lymphatic system (18, 19). In contrast, hydrophilic drugs and medium-chain fatty acids (MCFA) are transported via the portal vein and undergo hepatic first-pass metabolism before entering the systemic circulation, significantly influencing their bioavailability. Chylomicron transport of drugs increases bioavailability and bioactivity in distal organs (18).

The pharmacokinetics of orally administered and endogenously generated NO_2_-FA is impacted by their electrophilic reactivity, intestinal metabolism, esterification into glycerolipids, the formation and dissociation of nitroalkene-thiol adducts, and analytical challenges associated with defining chemical reactivity and stability (20). Oral administration of 10-NO_2_-OA to male dogs was found preferentially esterified to TAGs in plasma, but this study did not characterize distribution to the lipoprotein particles (chylomicron vs. VLDL) (21). The outstanding questions that motivated the present work are: 1) Do sex-dependent differences impact the metabolism and transport of NO_2_-FA? 2) Do NO_2_-FA incorporate into chylomicrons to then be distributed via the lymphatic system or gain access to the systemic circulation through the portal vein?

Herein, we report that NO_2_-FA is mainly transported in chylomicrons and that the extensive intestinal metabolism is not sex-dependent. Importantly, 10-NO_2_-OA significantly influenced the lymph flow, TAG output, and chylomicron release. After oral gavage of ^3^H-triolein in mice, 10-NO_2_-OA was shown to significantly inhibit lipid absorption and the appearance of labeled TAGs in plasma. These effects were evident both in the presence and absence of poloxamer-407. Notably, oral administration of 10-NO_2_-OA in obese subjects reduced plasma TAG levels, suggesting a potential impact on dietary fat absorption (17). Overall, these findings provide new understanding of the mechanisms of NO_2_-FA transport and reveal a significant reduction of lipid absorption. These properties lend a new perspective to the potential impact of both the endogenous gastric generation and the therapeutic administration of NO_2_-FA on lipid trafficking.

## Materials and Methods

Synthesis of (E)-10-nitro-octadec-9-enoic acid (10-NO_2_-OA) and the isotopically labeled internal standards, (E)-10-nitro[^15^N]-octadec-9-enoic-15,15,16,16-[d_4_] acid (10-[^15^N]O_2_-[d_4_]OA) and (E)-10-nitro[^15^N]-octadecanoic-15,15,16,16-[d_4_] acid (10-[^15^N]O_2_-[d_4_]SA) was performed as previously described (21, 22). The chemical and isotopic purities of labeled standards were determined to be ≥ 95% and ≥ 99%, respectively, through NMR and HPLC-MS analyses. Triheptadecanoin was purchased from Nu-Chek Prep, Inc., while ^3^H-triolein was from American Radiolabeled Chemicals. The basic formulation of the emulsion used for the vehicle and 10-NO_2_-OA delivery in the rat cannulation studies is shown in Supplemental Table S2. The lipids present in the emulsion did not interfere with the analytical methods used to quantify the 10-NO_2_-OA. Unless specified otherwise, analytical grade chemicals were obtained from Sigma. Solvents utilized for extractions and mass spectrometric analyses were purchased from Burdick and Jackson.

### Animal studies

To evaluate sex-related differences in the systemic distribution of 10-NO_2_-OA, fasted male and female Sprague-Dawley rats (∼175–275 g, 10 weeks old, n = 9 per group, Harlan) were gavaged twice daily, about 6 h apart, with 15 mg/kg 10-NO_2_-OA dissolved in sesame oil (30 mg/kg/day). Animals were anesthetized by CO_2_ inhalation before blood collection, performed by retroorbital puncture at specified time points. To describe the PK of 10-NO_2_-OA in rat plasma and to minimize blood-sample withdrawal, a two–time point limited sampling strategy was applied. No more than 2 blood samples from each animal (∼0.75 ml) were taken. Blood samples were centrifuged, and the plasma was collected and stored at −80 °C until used. In vivo experiments were performed according to the protocols approved by the University of Pittsburgh Institutional Animal Care and Use Committee (IACUC).

To assess the lymphatic transport and the portal absorption of 10-NO_2_-OA, a conscious rat fistula model having a cannulated mesenteric lymph duct with portal vein sampling was performed at the Mouse Metabolic Phenotyping Center, University of Cincinnati, Ohio, as previously reported (23, 24). Briefly, fasted male adult Sprague-Dawley rats (∼300–350 g, n = 9–10 per group) were infused for an hour with 3 ml of emulsion vehicle formulation with or without 10-NO_2_-OA (10 mg/ml) using a duodenal catheter. Lymph was consistently collected on ice over 6 h, and blood samples from the portal vein were obtained every hour, with subsequent plasma separation. Then, the samples were stored at −80 °C until needed.

To assess the total dietary fat absorption and gastrointestinal distribution, C57BL/6J male mice (∼20–25 g, 10–12 weeks old, n = 4–6 per group, The Jackson lab) were fasted overnight. In the morning, mice were dosed i.p. with or without 1 mg/g poloxamer-407 (P407) (−1 h) to block all lipoprotein clearance. After one hour (0 h), mice were gavaged with 2 μCi ^3^H-triolein/100 μl olive oil/10 g with or without 2 mg/10 g 10-NO_2_-OA. Blood samples were collected on ice from the tail at −1, 0 h before and 1, 2, 3, 4, 5, 6 h after the gavage, plasma was separated by centrifugation and stored at −80 °C until needed. At 6 h, mice were euthanized by CO_2_ exposure and cervical dislocation and dissected for further analysis. All surgical procedures were approved by the University of Pittsburgh IACUC and comply with the NIH Guide for the Care and Use of Laboratory Animals.

### Analysis of free and esterified 10-NO_2_-OA in plasma and lymph

Levels of free 10-NO_2_-OA and its main metabolite 10-nitro-octadecanoic acid (10-nitro stearic acid, 10-NO_2_-SA) were determined by spiking 10 μl of plasma and lymph with 2 pmol of internal standard mixture 10-[^15^N]O_2_-[d_4_]OA/10-[^15^N]O_2_-[d_4_]SA. Lipids were extracted by adding 150 μl acetonitrile, followed by vortexing and centrifugation at 21,000 g for 10 min at 4 °C to remove precipitated proteins. The supernatant was then analyzed by HPLC-MS/MS.

The esterified 10-NO_2_-OA and 10-NO_2_-SA levels in plasma and lymph were analyzed using an acid hydrolysis method followed by HPLC-MS/MS quantification as previously (21). Briefly, 10 μl plasma and lymph fluid were spiked with 20 pmol internal standard mixture as mentioned above and incubated with 1 ml acetonitrile/HCl (9:1, v/v) at 90° C for 1 h, in the presence of 10 μl sulfanilamide (10% w/v acetonitrile) to prevent potential artifactual nitration. Then, 1 ml phosphate buffer 0.5 M pH 7.4 was added, followed by 2 ml hexane. The samples were vortexed and centrifuged at 2000 g for 5 min at 4 °C. Finally, the upper hexane phase was dried under a nitrogen stream, and lipids were dissolved in acetonitrile for HPLC-MS/MS analysis. The esterified levels were calculated by subtracting the concentration of free NO_2_-FA (pre-hydrolysis) from the total NO_2_-FA levels obtained after hydrolysis.

### Analysis of triglycerides and apolipoprotein B-48 levels and their secretion into rat lymph

Lymph samples (2.5 μl) were spiked with 530 pmol internal standard triheptadecanoin, and 200 μl ethyl acetate was added. Samples were vortexed, centrifuged for 5 min at 2000 g at 4 °C, and the supernatant diluted 10 times in ethyl acetate/acetonitrile (1:1, v/v). Analysis of TAGs was performed by HPLC-High Resolution (HR)-MS/MS evaluation. TAG levels were quantified by calculating the sum of peak area ratios (analyte/internal standard). To assess chylomicron output in rat lymph, an enzyme-linked immunosorbent assay (ELISA) kit (rat APOB ELISA, Biomatik) was used to measure apoB-48 levels in lymph samples. Lymph TAG and apoB-48 outputs were calculated multiplying the correspondent amounts by the lymph flow rate.

### Total dietary fat absorption and gastrointestinal distribution after ^3^H-triolein oral gavage

^3^H-Lipids from the plasma of mice gavaged with ^3^H-triolein were measured by scintillation counting with an LS 6500 Liquid Scintillation Counter (Beckman Coulter) as disintegrations per minute (DPM). The TAG concentrations were determined using a commercial kit (Randox Laboratories) and following the manufacturer’s instructions. In the mice group not pre-treated with P407, the small intestine was carefully further divided into three equal-length segments corresponding to the duodenum, jejunum, and ileum (labeled S1–3). The luminal contents of the three segments were collected by washing with 4 mL cold PBS after each intestinal segment was opened longitudinally, placed flat on a 60 mm Petri dish on ice, and then its mucosa was scraped and collected into 4 mL of cold PBS. Lipids from the entire stomach, as well as from the luminal and mucosal contents of the duodenum, jejunum, and ileum, along with the entire cecum and colon, were extracted using the Folch method (25). Briefly, the luminal and mucosal content and tissues were stored in chloroform/methanol (2:1) at 4 °C after collection. For Folch lipid extraction, samples were mixed and centrifuged at 350 g for 30 min at 4 °C. The bottom clear layers were transferred to clean glass tubes, which were then placed on a N-EVAP nitrogen evaporator (Organomation) with a water bath set at 55 °C to accelerate the drying process. Dried lipids were then resuspended in 400 μl of chloroform/methanol (2:1). An aliquot of the extracted lipid was used to determine the amount of ^3^H-lipid via liquid scintillation counting, and subsequently, the total amount of ^3^H-lipid in each tissue was calculated.

### Thin-layer chromatography analysis of intestinal lipids

Jejunal and ileal luminal and mucosal lipid extracts from mice not pre-treated with P407 were loaded onto activated silica gel TLC plates and fractionated using a solvent system of petroleum ether/diethyl ether/glacial acetic acid with a 25:5:1 vol ratio. Iodine vapor was used to stain the different lipid classes as well as the comigrating lipid standards. The spots corresponding to TAG, diacylglycerol (DAG), monoacylglycerol (MAG), FFA, and phospholipid (PL) were scraped into scintillation vials containing Ultima Gold XR scintillation fluid (PerkinElmer) for scintillation counting.

### HPLC-MS/MS analysis of nitroalkenes and triglycerides

Analysis of 10-NO_2_-OA and 10-NO_2_-SA was performed by HPLC-ESI-MS/MS using an analytical C18 Luna column (2 × 20 mm, 5 μm; Phenomenex) at a 0.7 ml/min flow rate, with a gradient solvent system consisting of water containing 0.1% acetic acid (solvent A) and acetonitrile containing 0.1% acetic acid (solvent B). Samples were chromatographically resolved using the following gradient program: 30%–100% solvent B (0–3 min); 100% solvent B (3–4 min) followed by 1 min re-equilibration at initial conditions. A QTRAP 6500+ triple quadrupole mass spectrometer (Sciex) was used in negative ion mode with the following parameters: declustering potential (DP) - 60 V, collision energy (CE) - 42 eV, entrance potential (EP) and collision cell exit potential (CXP) - 5 V, and source temperature of 650 °C. Quantitation of nitroalkenes was performed by stable isotopic dilution analysis using calibration curves and the following MRM transitions: 326.2/46 and 331.2/47 for 10-NO_2_-OA and its I.S. 10-[^15^N]O_2_-[d_4_]OA, respectively, and 328.2/46 and 333.2/47 for 10-NO_2_-SA and its I.S. 10-[^15^N]O_2_-[d_4_]SA, respectively. MRM transitions for all 10-NO_2_-OA and 10-NO_2_-SA beta-oxidized metabolites were also followed (16).

TAGs in lymph were analyzed by HPLC-HR-MS/MS using a C8 Luna column (2 × 150 mm, 5 μm, Phenomenex) with a flow rate of 0.4 ml/min. The mobile phases consisted of acetonitrile/water (9:1, v/v) 0.1% ammonium acetate (solvent A) and isopropanol/acetonitrile 0.1% ammonium acetate (7:3, v/v) (solvent B). The gradient program proceeded as follows: 35%–100% solvent B (0.1–10 min), 100% solvent B (10–13 min), and 4 min of re-equilibration to initial conditions. A Q-Exactive hybrid quadrupole-orbitrap mass spectrometer (ThermoFisher) was operated in positive mode with the following parameters: auxiliary gas heater temperature 250 °C, capillary temperature 300 °C, sheath gas flow rate 20, auxiliary gas flow rate 20, sweep gas flow rate 0, spray voltage 4 kV, and S-lens RF level 60 (%). The full mass scan analysis covered a range from 650 to 1400 m/z at a resolution of 17,500.

Lipidomics data were reported according to the Lipidomics Minimal Reporting Checklist (LSI) (Supplemental Data 1 and 2).

### Statistical analysis

PK data are expressed as means ± standard deviation (SD) and two-way ANOVA plus Sidak’s multiple comparisons were used for statistical significance. Area under the curve (AUC) are expressed as mean ± SD and statistic differences were obtained via unpaired *t* test analysis.

## Results

### Systemic distribution of nitroalkenes in male and female rats

**Fig. 1:**
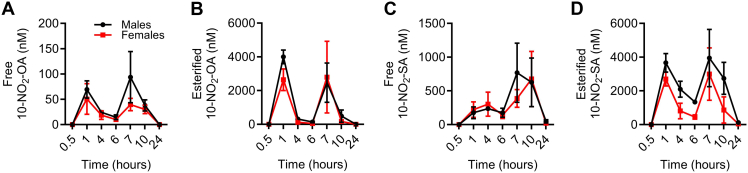
**Sex-related systemic distribution of orally administered 10-NO_2_-OA**. Plasma levels of (A) free and (B) esterified 10-NO_2_-OA, and its inactive metabolite (C) free and (D) esterified 10-NO_2_-SA, in male and female rats (n = 9 per group) gavaged twice, 6 h apart, with 15 mg/kg 10-NO_2_-OA. A two–time point limited sampling strategy was applied to minimize blood-sample withdrawal. Each time point represents the mean ± SD of one experiment with n = 3. No statistical differences were found between male and female rats by two ANOVA analyses.

By understanding how orally administered 10-NO_2_-OA is absorbed and distributed, critical PK/PD perspective is also provided for dietarily- and digestively generated NO_2_-FA species such as NO_2_-CLA. Since men and women may have different NO_2_-FA ADME profiles, we evaluated sex-related responses in rats for both the plasma levels and metabolism of 10-NO_2_-OA following 30 mg/kg/day oral administration. Both male and female rats exhibited comparable plasma concentrations of free and esterified 10-NO_2_-OA over 24 h (Fig. 1A, B). In agreement with a previous PK study in beagle dogs (21), 10-NO_2_-OA was preferentially esterified and reached micromolar plasma concentrations. After the administration of the first 15 mg/Kg dose, esterified 10-NO_2_-OA showed a C_max_ of 4 ± 0.4 μM at 1 h, ∼60 times greater than the free acid levels, followed by a ∼13-fold decrease at 4 h (Fig. 1A, B). After 6 h, a second 15 mg/Kg dose was administered, and the esterified levels were 2.5 ± 1.1 μM at 7 h, decreasing 5-fold at 10 h, and reaching basal levels of 5 nM ± 1.4 nM at 24 h. The primary metabolic inactivation of 10-NO_2_-OA was enzymatic reduction of the nitroalkene, yielding the non-electrophilic nitroalkane product 10-NO_2_-SA (26). 10-NO_2_-SA showed similar esterified plasma concentrations as 10-NO_2_-OA but significantly higher free levels, especially after a second dose. These levels peaked and remained at ∼650 nM at 7–10 h post-dosing, decreasing to ∼50 nM at 24 h (Fig. 1C, D).

### Intestinal absorption and metabolism of 10-NO_2_-OA

**Fig. 2:**
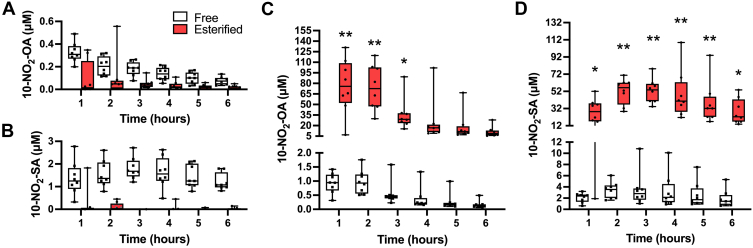
**Intestinal uptake and metabolism of 10-NO_2_-OA in a conscious rat fistula model**. Levels of NO_2_-FA in (A, B) portal plasma and (C, D) mesenteric lymph after duodenal infusion of 10-NO_2_-OA. 10-NO_2_-OA and 10-NO_2_-SA free acid and esterified forms are shown. Box and whiskers (minimum to maximum) plots are shown from a PK study (n = 8) with statistical significance assessed by two-way Anova (∗*P* < 0.005 and ∗∗*P* < 0.001).

To characterize the intestinal absorption of orally administered 10-NO_2_-OA, a portal vein and mesenteric lymph duct cannulated conscious rat model was employed (23). Portal vein sampling after duodenal administration of 10-NO_2_-OA showed low levels of free 10-NO_2_-OA and its metabolite 10-NO_2_-SA in plasma and low levels esterified in complex lipids (Fig. 2A, B). 10-NO_2_-OA achieved a peak plasma concentration of 0.3 ± 0.1 μM at 1 h, decreasing to 0.07 μM at 6 h, while 10-NO_2_-SA reached ∼1.2 μM at 1 h–6 h post-dosing. Analysis of lymph samples revealed that 10-NO_2_-OA was primarily esterified in chylomicron TAGs (Fig. 2C). Specifically, esterified 10-NO_2_-OA concentrations in lymph were ∼80 times greater than free acid levels and 245 times more concentrated than portal vein plasma levels, reaching ∼76 ± 39 μM in the first 2 h, decreasing to ∼11 ± 7 μM at 6 h. The concentration of esterified 10-NO_2_-SA in lymph was 3-fold lower than 10-NO_2_-OA at 1 h (Fig. 2D), increasing to 52 ± 14 μM at 2–3 h and reaching 28 ± 14 μM at 6 h. These data indicated that enterocyte uptake and incorporation into chylomicrons play an important role in oral 10-NO_2_-OA absorption and systemic distribution, thus avoiding hepatic first-pass metabolism.

### Incorporation of 10-NO_2_-OA into lymph triglycerides

Enterocytes absorb dietary fatty acids from the intestinal lumen, swiftly converting them with high yield into TAGs. Esterified products are then assembled into chylomicrons and secreted into the mesenteric lymphatics to reach the subclavian vein through the thoracic duct. The chylomicron core primarily consists of TAGs (85%–92%) (27). To assess the incorporation of fatty acid nitroalkenes into chylomicron TAGs, organic extracts of lymphatic fluid from rats supplemented with 10-NO_2_-OA were analyzed by HPLC-HR-MS/MS. MS analysis (at the 2 ppm level) revealed the presence of odd mass ions corresponding to ammoniated TAGs containing 10-NO_2_-OA [10-NO_2_-OA-TAG + NH_4_]^+^ (Supplemental Table S1). Three TAG species (*m/z* 945.78, 943.77, and 919.76) were previously identified in the plasma of dogs following oral administration 10-NO_2_-OA, with the incorporated fatty acids corresponding to a total number of carbons and double bonds of 54:4, 54:5 and 52:3 respectively (21). MS2 fragmentation, elemental composition analysis, and detection of FA chain-specific fragments unequivocally confirmed the molecular species level of TAG. Specifically, the presence or absence of fatty acid nitroalkenes leads to the generation of even- and odd-mass fragments. These structurally informative fragments correspond to a neutral loss of a fatty acid and ammonia [M-FA-NH_3_]^+^ (even mass for NO_2_-FA acid-containing TAGs), while the characteristic neutral loss of 344 amu corresponds to the loss of 10-NO_2_-OA and ammonia [M-(10-NO_2_-OA)-NH_3_]^+^. Other significant TAGs incorporating 10-NO_2_-OA displayed odd mass ions of *m/z* 837.69, 835.67, and 811.67, along with fatty acids totaling 46:2, 46:3, and 44:1 carbons and double bonds, respectively. MS2 analysis also showed fragments resulting from neutral losses of 189 amu corresponding to the medium-chain decanoic acid and ammonia [M-10:0-NH_3_]^+^.

### 10-NO_2_-OA modulates lymphatic flow, triglyceride absorption, and chylomicron secretion

**Fig. 3:**
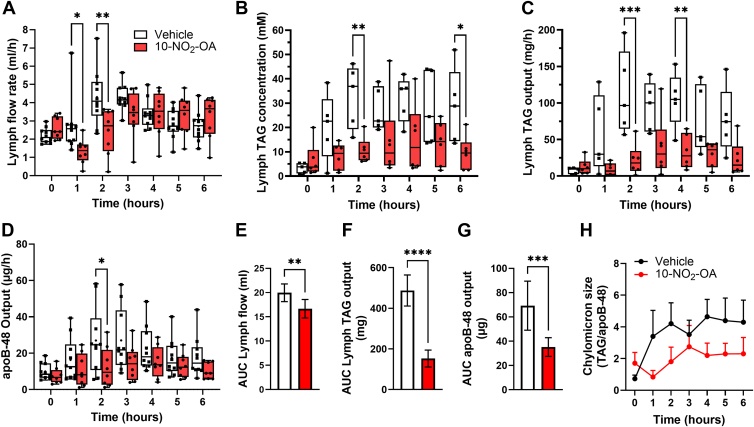
**10-NO_2_-OA inhibits lymph flow and chylomicron secretion**. A: Modulation of lymph flow rate following duodenal administration of vehicle (n = 10) and 10-NO_2_-OA (n = 8) in the lymph fistula rat model. Time-dependent changes in lymphatic (B) triglyceride levels, (C) triglyceride secretion, and (D) apoB-48 output following duodenal administration of vehicle (n = 5) and 10-NO_2_-OA (n = 6). Box and whiskers (minimum to maximum) plots from a single PK study are shown. Statistical significance between vehicle and 10-NO_2_-OA groups in the lymph flow rate and apoB-48 output was obtained via two-way ANOVA and was significant with ∗*P* < 0.05, ∗∗*P* < 0.005, and ∗∗∗*P* < 0.0005. Further differences for (E) lymph flow rate, and both (F) triglyceride and (G) apoB-48 outputs are shown via AUC (mean ± SD) and unpaired *t* test analysis (∗∗∗*P* < 0.0005, ∗∗∗∗*P* < 0.0001). H: Lymphatic chylomicron size is shown as the ratio of triglyceride to apoB-48 output and reported as mean ± SE.

**Supplemental Figure S1:**
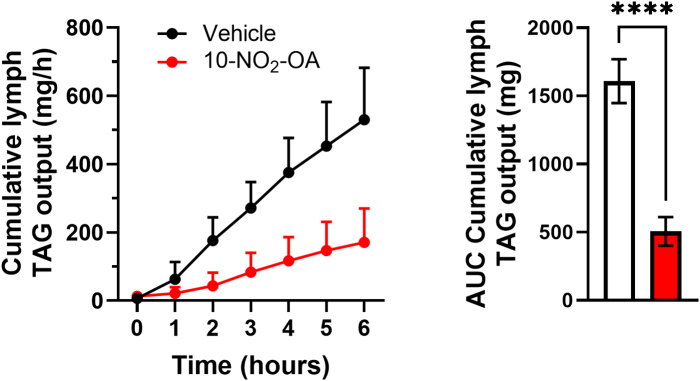
10-NO_2_-OA reduces cumulative lymph TAG output. Time dependent changes in cumulative lymphatic triglyceride secretion following duodenal administration of vehicle (n = 5) and 10-NO_2_-OA (n = 6) in the lymph fistula rat model. Data are shown as mean ± SD. Significant differences are shown via AUC (mean ± SD) and unpaired *t*-test analysis (∗∗∗∗*P* < 0.0001).

Lymphatic TAG-rich chylomicron transport is strongly affected by intestinal lymph flow, a process regulated by various factors such as the rhythmical contraction of smooth muscle cells lining the lymphatic walls, the intraluminal valve system, secretin and steroid hormones, among others (28). Thus, we evaluated the impact of 10-NO_2_-OA on lymph flow and TAG output of duodenal infusions and characterized the TAG profile. Administration of a vehicle emulsion for 1 h caused a gradual increase in lymph flow from 2.2 ± 0.4 ml/h during fasting to 4.3 ± 1.5 ml/h at 2–3 h to slowly decrease to 2.7 ± 0.8 ml/h at 6 h (Fig. 3A). In contrast, a 1-h infusion of a 10-NO_2_-OA fortified emulsion caused a significant decrease in lymph flow rate from 2.6 ± 0.6 ml/h during fasting to 1.3 ± 0.6 ml/h at 1 h, slowly increasing to 2.4 ± 1.2 ml/h at 2 h and normalizing at 4 h (infusion was switched to saline after 1 h). We next measured the concentration of TAGs in the collected lymph fluid, showing a sharp decrease after 10-NO_2_-OA administration and throughout the length of the study, despite the recovery of the lymph flow at the end of the study. This indicated a decoupling of lymph flow and chylomicron release (Fig. 3B). We next measured the total TAG output, which showed that 10-NO_2_-OA significantly inhibits TAG secretion into the lymphatics (Fig. 3C), aligning with the observed reduction in the cumulative TAG output (Supplemental Fig. S1). Area under the curve (AUC) analysis showed a significant reduction in the lymph fluid volume (Fig. 3E) and total TAG output (∼70%) (Fig. 3F) over the 6 h.

To assess whether reduced lymph flow and TAG output were associated with decreased chylomicron release into the mesenteric lymph, we quantified lymphatic fluid apoB-48. This apolipoprotein is essential for chylomicron assembly and secretion and is a reliable marker of chylomicron synthesis and release (29). As expected from changes in TAG output, 10-NO_2_-OA also significantly reduced apoB-48 release at 2 h, remaining low afterward (Fig. 3D). An area under the curve (AUC) analysis showed a significant reduction of apoB-48 release (Fig. 3G). We next estimated chylomicron size changes by evaluating the TAG load per apoB-48. 10-NO_2_-OA reduced the size of the released chylomicrons, affirming that NO_2_-FA exerted a complex regulation of fat absorption and transit into the lymphatics (Fig. 3H).

**Supplemental Figure S2:**
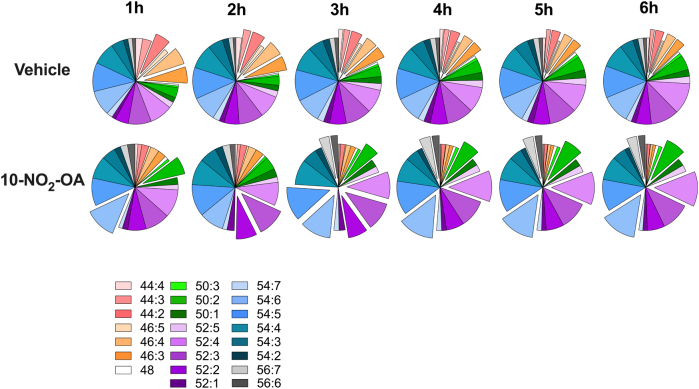
10-NO_2_-OA administration affects triacylglycerol composition of lymph chylomicrons. Time-dependent alteration in the composition of triglyceride chylomicrons, following duodenal administration of vehicle and 10-NO_2_-OA. Data are presented as percentages from a single PK study. Significant differences (*P* < 0.05) between the vehicle (n = 5) and the 10-NO2-OA group (n = 6) are shown by exploded slices.

Upon absorption by enterocytes, medium-chain fatty acids (MCFA) predominantly utilize the portal vein route, undergoing extensive hepatic metabolism before entering the systemic circulation. In the present study, duodenal administration of vehicle containing equal concentrations of long- and medium-chain TAGs, led to a significant accumulation (∼26%) of the latter, such as 44:4, 44:3, 44:2, 46:4, and 46:3 species, within lymphatic chylomicrons during the first 2 h, in comparison with the 10-NO_2_-OA-supplemented group (Supplemental Fig. S2). This enrichment subsided to ∼15% at later times. 10-NO_2_-OA administration also induced a significant alteration of the lymph TAG profile compared to the vehicle group, characterized by lower amounts of MCFA incorporated into TAGs (∼10% at 1h to ∼4% at 6 h) and by enrichment of TAG containing long-chain fatty acids (LCFA) such as 56:7, 56:6, 54:6, 54:5, 52:4, 52:3, 52:2, and 50:2, especially 3–6 h after oral dosing.

### 10-NO_2_-OA inhibits intestinal absorption and distribution of dietary fat in mice gavaged with ^3^H-triolein

**Fig. 4:**
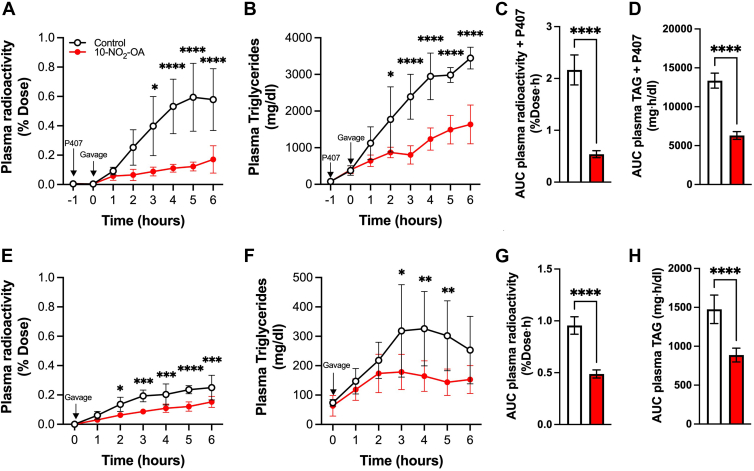
**10-NO_2_-OA inhibits dietary fat absorption and transit into systemic circulation**. Oral lipid tolerance test in mice following ^3^H-triolein gavage (0 h) (A–D) with or (E–H) without poloxamer-407 (P407) pre-treatment (−1 h). A, E: Plasma radioactivity was measured via scintillation counting and (B, F) plasma triglyceride concentrations quantified by LC-MS/MS. C, D, G, H: Statistical analysis of AUCs (mean ± SD) of plasma radioactivity and TAG levels was performed by unpaired *t* test analysis (∗∗∗∗*P* < 0.0001) between groups gavaged with Control and 10-NO_2_-OA with respective sample sizes of n = 4–5 (with P407) and n = 5–6 (without P407). PK data represent the mean ± SD and statistical differences were obtained via two-way Anova and significant with ∗*P* < 0.05, ∗∗*P* < 0.005, ∗∗∗*P* < 0.0005, ∗∗∗∗*P* < 0.0001.

To evaluate if 10-NO_2_-OA impairs fat absorption in vivo, we performed two additional studies in mice. First, we assessed the impact of 10-NO_2_-OA on dietary fat absorption following ^3^H-triolein oral gavage of mice pre-treated with poloxamer 407 (P407), to inhibit endothelial lipase and chylomicron degradation. Oral 10-NO_2_-OA significantly reduced plasma radioactivity and TAG levels (Fig. 4A,B), confirming a significant inhibition of dietary TAG absorption, consistent with the rat lymph data. 10-NO_2_-OA also inhibited the net levels of radiolabeled plasma lipids (AUC, Fig. 4C) and plasma TAG (AUC, Fig. 4D) by ∼75% and ∼50%, respectively. These results were also consistent with the data obtained in the lymph fistula model. We then performed a similar experiment in the absence of P407. While we observed a lower level of plasma radioactivity and plasma TAG that reflect the tissue extraction catalyzed by endothelial lipase, both levels and AUC in the 10-NO_2_-OA-treated mice remained significantly low (Fig. 4 E–H).

**Fig. 5:**
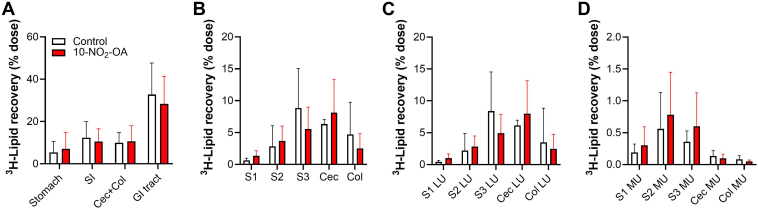
**Impact of 10-NO_2_-OA on the gastrointestinal distribution of dietary fat**. Distribution of dietary ^3^H-triolein radioactivity in mice not pre-treated with P407 is shown for the (A) gastrointestinal tract and (B) individual isolated segments, as a percentage of the administered dose. Percent of ^3^H recovered in the (C) lumen and (D) mucosa of intestine segments: duodenum (S1), jejunum (S2), ileum (S3), cecum, and colon. Data represent the mean ± SD from a single study. No statistical differences between the Control (n = 5) and the 10-NO_2_-OA group (n = 6) were obtained via two-way ANOVA analysis.

Next, we investigated the site for 10-NO_2_-OA inhibition of gastrointestinal tract ^3^H-triolein uptake in mice without P407 pre-treatment. While both control and 10-NO_2_-OA treated mice showed small amounts of radioactivity in the stomach, the small intestine (SI) and the cecum + colon showed higher levels without significant differences among groups (Fig. 5A). Further analysis of the SI segments revealed a trend toward lipid accumulation in duodenum (S1) and jejunum (S2) of 10-NO_2_-OA-treated group (Fig. 5B). Tracking radioactivity in isolated luminal (unabsorbed) and mucosal (absorbed) sections showed no notable differences in the lumen (Fig. 5C). However, the mucosa of the proximal intestine (duodenum, jejunum and ileum) showed a trend towards increased lipid accumulation in the 10-NO_2_-OA-treated group, consistent with an impaired transit to the lymphatics (Fig. 5D). As expected, cecum and colon mucosal levels were very low. Finally, TLC and liquid scintillation counting analysis showed no differences in the distribution of radioactivity among lipid species in either jejunal and ileal luminal or mucosal samples (data not shown).

## Discussion

The electrophilic small-molecule nitroalkene 10-NO_2_-OA exhibits unique PK/PD profiles and has been used as a biosimilar for endogenous nitro-conjugated linoleic acid (NO_2_-CLA) in therapeutic development programs. By virtue of its electrophilic character, 10-NO_2_-OA reversibly reacts with hyper-reactive cysteine thiolates in proteins and glutathione (GSH), leading to post-translational protein modifications (PTM), the downstream modulation of multiple signaling and metabolic responses and the eventual cellular elimination of GS-nitroalkene adducts via the multidrug resistance protein-1 transporter (30). Specifically, 10-NO_2_-OA activates the Nrf2 and heat shock response signaling, thus promoting antioxidant defenses, cytoprotective and tissue repair responses (31, 32). In addition, it inhibits NF-κB, the stimulator of interferon genes (STING), calcineurin, and soluble epoxy hydrolase (sEH), among others (33, 34, 35, 36, 37), collectively suppressing pro-inflammatory cytokine and adhesion molecule expression and promoting adaptive responses. Murine models of a) hepatic steatosis (38), b) abdominal wall defects (39), c) lung injury and fibrosis (40, 41), d) sickle cell disease (42), and e) Parkinson’s disease (15), among others, all respond beneficially to 10-NO_2_-OA administration. These multi-target signaling and tissue-protective actions have motivated the clinical translation of this class of mediators. Extensive preclinical toxicology, PK, and Phase 1 human studies support the safety and signaling pathway engagement of 10-NO_2_-OA (17). This work has been extended to an ongoing Phase 2 trial of the therapeutic potential of 10-NO_2_-OA in treating airway hyperreactivity in obesity-related asthma (NCT03762395).

Despite these findings, a comprehensive PK analysis of fatty acid nitroalkene derivatives after gastric formation or oral administration is lacking. Specifically, our understanding of absorption mechanisms, potential sex-related differences and impact on fat trafficking within the intestine and systemically is incomplete and crucial for derisking therapeutic strategies and understanding the physiological impact of endogenous inflammatory and digestive formation of NO_2_-CLA. The preferential incorporation of nitroalkenes into TAG in mice after oral administration suggested lymphatic transport as a key mechanism of intestinal absorption (9, 21, 43, 44). Herein, we used a conscious rat lymph fistula model and established lymphatic transport as the primary mechanism of intestinal absorption that showed no sex differences in initial metabolism and transport. A significant new finding was that 10-NO_2_-OA potently modulates lipid trafficking via several parameters of fat absorption, lymph flow, TAG concentration and output, apoB-48 release, and chylomicron composition and size. These results reveal a new level of regulation of fat absorption and transport by small molecule nitroalkenes and fill gaps in the understanding of nitroalkene PK by defining new regulatory mechanisms.

Previous radiochromatographic analysis confirmed the intestinal absorption of 10-NO_2_-OA, with 35% of the radioactivity excreted in urine and feces representing the largest excretion pool (16). It has not been established yet if fecal excretion corresponded to unabsorbed material or was excreted as part of the enterohepatic circulation. Data from Phase 1 clinical trials of orally administered 10-NO_2_-OA reported low nM plasma concentrations of the free acid 10-NO_2_-OA. These initial studies did not address other loci of fatty acid nitroalkene distribution, such as complex lipid-esterified and protein-adducted species (17). With respect to protein binding, albumin was initially considered as a plasma transporter of 10-NO_2_-OA, due to a) ∼600 μM plasma albumin concentration, b) the nucleophilic Cys34 of albumin being the principal thiol in plasma, and c) an ability of albumin to potentially non-covalently bind up to 7 molecules of 10-NO_2_-OA (45). It is now understood that albumin Cys34 does not react with NO_2_-OA (45, 46). The present data reveal that albumin only plays a minor role in the distribution of orally administered fatty acid nitroalkenes, with low levels of plasma “free” 10-NO_2_-OA when compared with esterified species. Nonetheless, albumin might play a preponderant role in the biodistribution of intravenously administered 10-NO_2_-OA, a possibility that has not yet been investigated.

Sex differences in the gastrointestinal tract have been reported, with gastric emptying time and expression of certain gastric enzymes, such as alcohol dehydrogenase, being different between males and females (47). 10-NO_2_-OA is largely metabolized by prostaglandin reductase 1 (*PTGR1*) to its inactive reduced metabolite 10-NO_2_-SA (26), whereas its corresponding non-nitrated dietary fatty acid, oleic acid, is not a substrate for this enzyme. Since the possible sex-dependent expression of *PTGR1* could significantly influence 10-NO_2_-OA plasma levels, we analyzed free and esterified 10-NO_2_-OA and 10-NO_2_-SA in both male and female rats. Notably, the presence of the reduced inactive metabolite 10-NO_2_-SA in lymph TAGs confirms a pre-systemic metabolism by intestinal *PTGR1*, highly expressed in enterocytes (48). In agreement with a previous PK study in dogs, 10-NO_2_-OA was preferentially esterified in plasma lipids (21) and showed no significant sex-based differences in intestinal absorption. Although we did not evaluate female rats using the conscious lymph fistula model, this result aligns with previous observations in a similar model that showed comparable intestinal absorption and lymphatic transport of dietary lipids in females (during periods of low estrogen plasma concentrations) and males (49).

The physiological characteristics of the digestive system (50) and the physiochemical properties of 10-NO_2_-OA dictated its intestinal absorption upon oral administration and subsequent systemic availability. At the intestinal level, the enteric epithelium establishes a substantial barrier, limiting access to the systemic circulation. Factors such as solubility, permeability, enterocyte metabolism and both portal and lymphatic transport all play an important role in absorption. 10-NO_2_-OA possesses physiochemical properties promoting passive diffusion into enterocytes and transport by the lymphatics, including: (a) a partition coefficient of logP >5, (b) a high long-chain TAG solubility of >50 mg/g, and (c) a molecular weight (MW) < 500 (18). Furthermore, 10-NO_2_-OA can also be transported by fatty acid transport proteins such as CD36 (51), and is esterified by dietary 2-monoacylglycerols within enterocytes, leading to the generation of TAGs which are then incorporated into lipid droplets to become mature TAG-enriched chylomicrons (52).

Intestinal lymph flow is regulated by hormones, smooth muscle fibers, and lymphatic valves, ensuring efficient systemic distribution of TAG-rich chylomicrons to tissues (53, 54). Notably, a marked delay in chylomicron appearance occurs when the lymph flow falls below 30–40 μl/min (55). The infusion of 10-NO_2_-OA within the initial 2 h significantly reduced the lymph flow to 22.16 μl/min, impacting lymphatic TAG and apoB-48 outputs. In the same conscious lymph fistula rat model, glucagon-like peptide-1 (GLP-1) reduces intestinal lymph flow and TAG absorption via the glucagon-like peptide-1 receptor (GLP-1r) (24). This receptor contains a Cys347 that reacts with multiple electrophiles via Michael addition to enhance GLP-1r signaling (56). Further studies may reveal if 10-NO_2_-OA modulates GLP-1r signaling through nitroalkylation, thus promoting decreased lymph flow and TAG uptake by enterocytes.

^3^H-triolein tracking is a valuable method to quantify the molecular physiology of intestinal fat absorption, including luminal digestion, enterocyte processing and secretion into circulation. Reduced systemic radioactivity and decreased TAG levels in mice treated with 10-NO_2_-OA confirmed the inhibition of fat absorption, consistent with observed reduced lymphatic flow and chylomicron output in rats. These results align with clinical data from obese males, orally treated with 10-NO_2_-OA. These subjects displayed significantly lower plasma TAG concentrations in comparison with the placebo group (17). The decreased radiolabeled TAG levels in plasma were not dependent on increased endothelial lipase activity, as demonstrated by a lack of impact by the lipoprotein lipase inhibitor P407. Intestinal lumen radioactivity determinations did not reveal differences between the vehicle and 10-NO_2_-OA treated animals. Analysis of mucosa showed a trend of increased lipid accumulation in the 10-NO_2_-OA treated group, consistent with a decreased transit into the lymphatic system. Although we were able to quantify labeled TAG, diacylglycerols, monoacylglycerols, phospholipids, and fatty acids in the lumen and mucosa of the intestine, we did not observe significant changes to these species that would explain the significant decrease in the appearance of TAG in lymph in 10-NO_2_-OA treated animals. This suggests that changes in these species through enzymatic inhibition of lipases or acyltransferases (*MGAT* and *DGAT*) could occur, or chylomicron loading and maturation (through the regulation of microsomal triglyceride transfer protein or apoB expression) are occurring but were not evident by our analyses. These are logical future studies and will require studying fat absorption across multiple time points after 10-NO_2_-OA treatment. Nonetheless, 10-NO_2_-OA treatment led to a significant accumulation of TAG-containing long-chain fatty acids (LCFA) in the lymph fistula model, modifying the chylomicron TAG profile. This HPLC-MS/MS analysis is sensitive to modest quantitative differences, as opposed to radioisotope tracing, and suggested a potential modulatory action of 10-NO_2_-OA on the monoglyceride pathway. While rodents express two *MGAT* isoforms, only monoacylglycerol acyltransferase 2 (*MGAT2*) is highly expressed in the small intestine and displays a higher affinity for LCFA than MCFA (57, 58). Interestingly, mutations within the catalytic domain of *MGAT2*, specifically at Cys334, enhance its enzymatic activity (59). Future investigations may reveal whether 10-NO_2_-OA induces the PTM of this functionally significant *MGAT2*-Cys334 to promote increased LCFA esterification within lymphatic TAGs.

This study advances our understanding of the absorption, metabolism, and physiological impact of orally administered NO_2_-FA and provides a foundation for comprehending the actions of digestively generated lipid nitroalkene and nitro-nitrate species. We confirmed that 10-NO_2_-OA is primarily absorbed and transported via the lymphatic system as esterified TAG species, with initial metabolism occurring in enterocytes independent of sex. Notably, 10-NO_2_-OA significantly modulated key aspects of lipid absorption and transport, including lymph flow, chylomicron output, and TAG composition, highlighting a novel regulatory role for electrophilic lipids in intestinal lipid handling. These findings, combined with known multi-target anti-inflammatory and cytoprotective actions of nitroalkenes, expand our understanding of the beneficial physiological actions of these species and support further exploration of gut-localized effects and systemic actions following oral administration or gastric formation of electrophilic fatty acids. This work also fills a critical gap in understanding nitroalkene pharmacology, with the significant decrease in TAG absorption opening new avenues for optimization as orally bioavailable agents targeting cardiometabolic and inflammatory diseases.

## Data availability

The datasets generated during and/or analyzed during the current study are available from the corresponding author upon request.

## Supplemental data

This article contains supplemental data.

## Conflict of interest

The authors declare the following financial interests/personal relationships which may be considered as potential competing interests: F. J. S., B. A. F. and M. F. acknowledge financial interest in Creegh Pharmaceuticals, Inc. F. J. S. has financial interest in Furanica Inc.

## Supplemental data

Supplemental Table S1 Characterization of the most abundant lymphatic NO_2_-FA-containing TAGs as ammonium adducts by HPLC-HR-MS/MS analysis

Supplemental Table S2 Emulsion formulation ingredients for the vehicle and 10-NO_2_-OA delivery in rat cannulation studies

Supplemental Data 1 Lipidomics Minimal Reporting Checklist (LSI).

Supplemental Data 2 Lipidomics Minimal Reporting Checklist (LSI).
