# Supplementary material for: Fatty acid nitroalkenes regulate intestinal lipid absorption
Source: J Lipid Res. 2025 Jul 4;66(8):100855. doi: 10.1016/j.jlr.2025.100855 (PMC12341606; doi:10.1016/j.jlr.2025.100855)
Supplement: Supplemental Table S1 — Characterization of the most abundant lymphatic NO2-FA-containing TAGs as ammonium adducts by HPLC-HR-MS/MS analysis [file mmc1.pdf]

Ammoniated TG	Molecular species level	Fragment ions	Elemental composition	Experimental mass	Theoretical mass	Retention time (min)
TG 54:4;NO_2	TG 18:1;NO_2_18:1_18:2		+$C_{57}H_{105}N_2O_8$	945.7837	945.7865	5.45
		-FA 18:2(+HO)-TG(17)	+$C_{39}H_{70}NO_6$	648.5184	648.5198	
		-FA 18:1(+HO)-TG(17)	+$C_{39}H_{68}NO_6$	646.5035	646.5041	
		-FA 18:1;NO_2(+HO)-TG(17)	+$C_{39}H_{69}O_4$	601.5179	601.519	
	TG 18:0;NO_2_18:2_18:2		+$C_{57}H_{105}N_2O_8$	945.7837	945.7865	5.45
		-FA 18:2(+HO)-TG(17)	+$C_{39}H_{70}NO_6$	648.5184	648.5198	
		-FA 18:0;NO_2(+HO)-TG(17)	+$C_{39}H_{67}O_4$	599.5037	599.5034	
TG 54:5;NO_2	TG 18:1;NO_2_18:2_18:2		+$C_{57}H_{103}N_2O_8$	943.7719	943.7709	4.94
		-FA 18:2(+HO)-TG(17)	+$C_{39}H_{68}NO_6$	646.5045	646.5041	
		-FA 18:1;NO_2(+HO)-TG(17)	+$C_{39}H_{67}O_4$	599.5009	599.5034	
TG 54:6;NO_2	TG 18:1;NO_2_18:2_18:3		+$C_{57}H_{101}N_2O_8$	941.7549	941.7552	4.53
		-FA 18:3(+HO)-TG(17)	+$C_{39}H_{68}NO_6$	646.5036	646.5041	
		-FA 18:2(+HO)-TG(17)	+$C_{39}H_{66}NO_6$	644.489	644.4885	
		-FA 18:1;NO_2(+HO)-TG(17)	+$C_{39}H_{65}O_4$	597.4855	597.4877	
TG 52:3;NO_2	TG 18:1;NO_2_16:0_18:2		+$C_{55}H_{103}N_2O_8$	919.7679	919.7709	5.35
		-FA 16:0(+HO)-TG(17)	+$C_{39}H_{68}NO_6$	646.5026	646.5041	
		-FA 18:2(+HO)-TG(17)	+$C_{37}H_{68}NO_6$	622.5048	622.5041	
		-FA 18:1;NO_2(+HO)-TG(17)	+$C_{37}H_{67}O_4$	575.5037	575.5034	
TG 46:2;NO_2	TG 18:1;NO_2_10:0_18:1		+$C_{49}H_{93}N_2O_8$	837.6911	837.6926	4.52
		-FA 10:0(+HO)-TG(17)	+$C_{39}H_{70}NO_6$	648.5187	648.5198	
		-FA 18:1(+HO)-TG(17)	+$C_{31}H_{56}NO_6$	538.4081	538.4102	
		-FA 18:1;NO_2(+HO)-TG(17)	+$C_{31}H_{57}O_4$	493.4245	493.4251	
TG 46:3;NO_2	TG 18:1;NO_2_10:0_18:2		+$C_{49}H_{91}N_2O_8$	835.6775	835.677	4.08
		-FA 10:0(+HO)-TG(17)	+$C_{39}H_{68}NO_6$	646.502	646.5041	
		-FA 18:2(+HO)-TG(17)	+$C_{31}H_{56}NO_6$	538.4092	538.4102	
		-FA 18:1;NO_2(+HO)-TG(17)	+$C_{31}H_{55}O_4$	491.409	491.4095	
TG 44:1;NO_2	TG 18:1;NO_2_10:0_16:0		+$C_{47}H_{91}N_2O_8$	811.6755	811.677	4.51
		-FA 10:0(+HO)-TG(17)	+$C_{37}H_{68}NO_6$	622.506	622.5041	
		-FA 16:0(+HO)-TG(17)	+$C_{31}H_{56}NO_6$	538.4089	538.4102	
		-FA 18:1;NO_2(+HO)-TG(17)	+$C_{29}H_{55}O_4$	467.4088	467.4095	
