# Supplementary material for: Fatty acid nitroalkenes regulate intestinal lipid absorption
Source: J Lipid Res. 2025 Jul 4;66(8):100855. doi: 10.1016/j.jlr.2025.100855 (PMC12341606; doi:10.1016/j.jlr.2025.100855)
Supplement: Supplemental Table S2 — Emulsion formulation ingredients for the vehicle and 10-NO2-OA delivery in rat cannulation studies [file mmc2.pdf]

Ingredient	Purpose	mg/ml	% (w/v)
Soybean Oil, USP<br>CAS number 8001-22-7	Solubilizer	50	5
Miglyol812, NF<br>CAS number 37332-31-3	Solubilizer	50	5
Egg Phospholipids,<br>CAS number 8002-43-5	Emulsifier	50	5
Sucrose, NF<br>CAS number 57-50-1	Stabilizer	100	10
Edetate Disodium, USP<br>CAS number 6381-92-6	Antioxidant	0.155	0.0155
Sodium Hydroxide, NF or<br>Hydrochloric Acid, NF	pH adjust	q.s.	q.s.
Sterile Water for injection	Diluent	q.s.	q.s.

q.s. = quantum sufficit
