# Supplementary material for: Fatty acid nitroalkenes regulate intestinal lipid absorption
Source: J Lipid Res. 2025 Jul 4;66(8):100855. doi: 10.1016/j.jlr.2025.100855 (PMC12341606; doi:10.1016/j.jlr.2025.100855)
Supplement: Supplemental Data 1 — Lipidomics Minimal Reporting Checklist (LSI). [file mmc3.pdf]

Created by https://lipidomicstandards.org, version v2.4.0

Contents of Report

Separation Workflow 1
Overall study design . . . 1
Lipid extraction . . . 1
Analytical platform . . . 1
Quality control . . . 2
Method qualification and validation . . . 2
Reporting . . . 2

Sample Descriptions 2
Analysis of free and esterified nitroalkenes in rat plasma / rat / Plasma . . . 2

Lipid Class Descriptions 3
1) FA[M-H]- / Lipid identification . . . 3
1) FA[M-H]- / Lipid quantification . . . 3

Separation Workflow

Overall study design

Title of the study	Intestinal absorption of an orally administered electrophilic small molecule nitroalkene - Analysis of FA 18:1;NO2 and FA 18:0;NO2		
Document creation date	08/02/2024	Corresponding Email	maf167@pitt.edu
Principal investigator	Marco Fazzari	Is the workflow targeted or untargeted?	Targeted
Institution	University of Pittsburgh	Clinical	No

Lipid extraction

Extraction method	1-phase system	Were internal standards added prior extraction?	Yes
pH adjustment	None	Special conditions	-
1-phase system	Acetonitrile	Derivatization	-

Analytical platform

Ionizaton additives	Acetic acid	Ion source	ESI
Number of separation dimensions	One dimension	MS Level	MS2
Separation type 1	LC	Mass window for precursor ion isolation (in Da total isolation window)	1
Separation mode 1 (liquid)	RP	Mass resolution for detected ion at MS2	Low resolution
Detector	Mass spectrometer	Resolution at MS2	Low
MS type	QTrap	Recording mode of raw data at MS2	Profile mode
MS vendor	SCIEX	Was/Were additional dimension/techniques used	No

Quality control

Blanks	Yes	Quality control	Yes
Type of Blanks	Extraction blank, Solvent blank, Internal standard blank	Type of QC sample	Sample pool

Method qualification and validation

Method validation	No

Reporting

Are reported raw data uploaded into repository?	No	Raw data upload	Available on request
Are metadata available?	Available on request	Additional comments	-

Sample Descriptions

Analysis of free and esterified nitroalkenes in rat plasma / rat / Plasma

Storage and collection conditions	Available	Additives	None
Provided preanalytical information	-	Were samples stored under inert gas?	No
Temperature handling original sample	4-8 °C	Additional preservation methods	No
Instant sample preparation	No	Biobank samples	No
Storage temperature	-80 °C		

Lipid Class Descriptions

1) FA[M-H]- / Lipid identification

Lipid class	FA	Limit of detection	S/N ratio > 3
MS Level for identification	MS2	RT verified by standard	Yes
Identification level	Species level	Separation of isobaric/isomeric interferece confirmed	Yes
Polarity mode	Negative	Model for separation prediction	No
Type of negative (precursor)ion	[M-H]-	Additional dimension/techniques	-
Fragments for identification		Lipid Identification Software	Analyst

Fragment name
46 (-NO2)

Isotope correction at MS2	No	Data manipulation	-
MS2 verified by standard	Yes	Nomenclature for intact lipid molecule	Yes
Background check at MS2	Yes	Nomenclature for fragment ions	Yes
Did you presume assumptions for identification?	No	Further identification remarks	-
Check on:	-		

1) FA[M-H]- / Lipid quantification

Quantitative	Yes	Type I isotope correction	No
MS Level for quantification	MS2	Limit of quantification	No
Internal lipid standard(s) MS2		Normalization to reference	No

Internal standard	Fragment(s)	Endogenous subclass
FA 18:1(9E)[(15,15,16,1 [15N]	47 (-[15N]O2)	FA
FA 18:0[(15,15,16,16)D4	47 (-[15N]O2)	FA

Type of quantification	Calibration line	Lipid Quantification Software	Analyst
Type of calibration line	Solvent based	Batch correction	No
Species calibration line	-	Further quantification remarks	-
Response correction	No		
