# Supplementary material for: Fatty acid nitroalkenes regulate intestinal lipid absorption
Source: J Lipid Res. 2025 Jul 4;66(8):100855. doi: 10.1016/j.jlr.2025.100855 (PMC12341606; doi:10.1016/j.jlr.2025.100855)
Supplement: Supplemental Data 2 — Lipidomics Minimal Reporting Checklist (LSI). [file mmc4.pdf]

Created by https://lipidomicstandards.org, version v2.4.0

Contents of Report

Separation Workflow

Overall study design

Title of the study	Intestinal absorption of an orally administered electrophilic small molecule nitroalkene - Analysis of TG		
Document creation date	08/02/2024	Corresponding Email	maf167@pitt.edu
Principal investigator	Marco Fazzari	Is the workflow targeted or untargeted?	Untargeted
Institution	University of Pittsburgh	Clinical	No

Lipid extraction

Extraction method	2-phase system	Were internal standards added prior extraction?	Yes
pH adjustment	None	Special conditions	-
2-phase system	Ethyl acetate	Derivatization	-

Analytical platform

Ionizaton additives	Ammonium acetate	Resolution at m/z 200 at MS1	17500
Number of separation dimensions	One dimension	Mass accuracy in ppm at MS1	2
Separation type 1	LC	Recording mode of raw data at MS1	Profile mode
Separation mode 1 (liquid)	RP	Mass window for precursor ion isolation (in Da total isolation window)	1
Detector	Mass spectrometer	Mass resolution for detected ion at MS2	High resolution
MS type	Orbitrap	Resolution at m/z 200 at MS2	35000
MS vendor	Thermo	Mass accuracy in ppm at MS2	2
Ion source	ESI	Recording mode of raw data at MS2	Profile mode
MS Level	MS1, MS2	Was/Were additional dimension/techniques used	No
Mass resolution for detected ion at MS1	High resolution		

Quality control

Blanks	Yes	Quality control	Yes
Type of Blanks	Extraction blank, Solvent blank, Internal standard blank	Type of QC sample	Sample pool

Method qualification and validation

Method validation	No

Reporting

Are reported raw data uploaded into repository?	No	Raw data upload	Available on request
Are metadata available?	Available on request	Additional comments	-

Sample Descriptions

Analysis of triglycerides in rat lymph / rat / Other liquid material

Storage and collection conditions	Available	Additives	None
Provided preanalytical information	-	Were samples stored under inert gas?	No
Temperature handling original sample	4-8 °C	Additional preservation methods	No
Instant sample preparation	No	Biobank samples	No
Storage temperature	-80 °C		

Lipid Class Descriptions

1) TG[M+NH4]+ / Lipid identification

Lipid class	TG	Did you presume assumptions for identification?	No
MS Level for identification	MS1, MS2	Check on:	-
Identification level	Molecular species level	Limit of detection	S/N ratio > 3
Polarity mode	Positive	RT verified by standard	Yes
Type of positive (precursor)ion	[M+NH4]+	Separation of isobaric/isomeric interferece confirmed	Yes
Fragments for identification<br>**Fragment name**<br>-FA(+HO)-(NH3)		Model for separation prediction	No
Isotope correction at MS1	No	Additional dimension/techniques	-
Isotope correction at MS2	No	Lipid Identification Software	Xcalibur
MS1 verified by standard	Yes	Data manipulation	-
MS2 verified by standard	Yes	Nomenclature for intact lipid molecule	Yes
Background check at MS1	Yes	Nomenclature for fragment ions	Yes
Background check at MS2	Yes	Further identification remarks	-

1) TG[M+NH4]+ / Lipid quantification

Quantitative	No	Batch correction	No
Normalization to reference	Yes	Further quantification remarks	-
